# Development of a novel anti-liver fibrosis formula with luteolin, licochalcone A, aloe-emodin and acacetin by network pharmacology and transcriptomics analysis

**DOI:** 10.1080/13880209.2021.1999275

**Published:** 2021-11-22

**Authors:** Yuan Zhou, Rong Wu, Fei-fei Cai, Wen-Jun Zhou, Yi-Yu Lu, Hui Zhang, Qi-Long Chen, Ming-Yu Sun, Shi-Bing Su

**Affiliations:** a Institute of Interdisciplinary Integrative Medicine Research, Shanghai University of Traditional Chinese Medicine, Shanghai, China; b Key Laboratory of Liver and Kidney Diseases (Ministry of Education), Institute of Liver Diseases, Shuguang Hospital, Shanghai University of Traditional Chinese Medicine, Shanghai, China

**Keywords:** Xiaoyaosan decoction, LLAAF, anti-liver fibrotic effect

## Abstract

**Context:**

Xiaoyaosan decoction (XYS), a classical Traditional Chinese Medicine (TCM) formula is used to treat liver fibrosis in clinics.

**Objective:**

This study explores defined compound combinations from XYS decoction to treat liver fibrosis.

**Materials and methods:**

Network pharmacology combined with transcriptomics analysis was used to analyze the XYS decoction and liver depression and spleen deficiency syndrome liver fibrosis. From the constructed XYS-Syndrome-liver fibrosis network, the top 10 active formulas were developed by topological analysis according to network stability. The most active formula was determined by *in vitro* study. The anti-fibrosis effect was evaluated by *in vitro* and *in vivo* studies.

**Results:**

According to the network XYS-Syndrome-liver fibrosis network, 8 key compounds and 255 combinations were predicted from in XYS. Luteolin, licochalcone A, aloe-emodin and acacetin formula (LLAAF) had a synergistic effect on the proliferation inhibition of hepatic stellate cells compared to individual compounds alone. The treatment of XYS and LLAAF showed a similar anti-liver fibrotic effect that reduced histopathological changes of liver fibrosis, Hyp content and levels of α-SMA and collagen I in CCl_4_-induced liver fibrosis in rats. Transcriptomics analysis revealed LLAAF regulated PI3K-Akt, AMPK, FoxO, Jak-STAT3, P53, cell cycle, focal adhesion, and PPAR signalling. Furthermore, LLAAF was confirmed to regulate Jak-STAT and PI3K-Akt-FoxO signalling *in vitro* and *in vivo*.

**Conclusions:**

This study developed a novel anti-liver formula LLAAF from XYS, and demonstrated its anti-liver fibrotic activity which may be involved in the regulation of Jak-STAT and PI3K-Akt-FoxO signalling.

## Introduction

Liver fibrosis is a pathological condition characterized by the replacement of normal liver tissue with scar tissue due to a variety of liver diseases, such as hepatitis B, hepatitis C, autoimmune liver diseases, as well as alcohol-induced or non-alcoholic fatty liver disease. It occurs due to repeated damage and repair processes, which are directly related to excessive accumulation of the extracellular matrix (Seki and Schwabe 2015). Excessive liver fibrosis can lead to hepatic cirrhosis and loss of liver function as well as digestive tract bleeding and hepatocellular carcinoma (Koyama and Brenner 2015). Liver fibrosis is involved in multiple liver cell types, including hepatic parenchymal cells, Kupffer cells, endothelial cells, hepatic stellate cells (HSCs), and infiltrated blood inflammatory cells, which proliferate and replace lost liver cells and produce the extracellular matrix in the injured liver (Higashi et al. 2017). Molecularly, upon the expression of liver disease-induced fibrogenic factors, such as transforming growth factor-β (TGF-β) as a profibrotic mediator, HSCs start to proliferate from their quiescent state due to the loss of cytoplasmic retinoids into proliferative and fibrogenic myofibroblasts, which express α-smooth muscle actin (α-SMA) and extracellular matrix type I, III and IV collagen, and depose them into the space of the lost hepatic parenchymal cells, resulting in liver fibrosis (Tsuchida and Friedman 2017). However, the molecular mechanisms of hepatic fibrogenesis remain to be defined, and there is still a lack of effective drugs for the treatment of liver fibrosis (Yoon et al. 2016).

Chinese herbal medicines have demonstrated anti-fibrotic effects *in vitro* and *in vivo* (Liu et al. 2009; Sanodiya et al. 2009; Tang et al. 2011; Dong and Su 2014; Zhang and Schuppan 2014). The advantage of Chinese herbal formulae (CHFs) is that the synergy of various herbs containing different active ingredients which can achieve an efficient treatment for human disease. However, a number of CHFs need to be precisely analyzed for their constituents and active compounds. According to traditional Chinese medicine (TCM), the treatment of patients is based on syndrome (ZHENG) differentiation (Su et al. 2014). For example, a subtype of liver fibrosis is referred to as liver depression and spleen deficiency syndrome (LDSDS) in TCM, and is often treated with Xiaoyaosan (XYS) (Liver Disease Committee 2019), a classic CHF derived from ‘Prescriptions of the People’s Welfare Pharmacy’ in the Song dynasty of China (960–1127 AD). It consists of *Bupleurum chinense* DC. (Apiaceae), *Angelica sinensis* (Oliv.) Diels (Apiaceae), *Paeonia lactiflora* Pall. (Paeoniaceae), *Atractylodes macrocephala* Koidz. (Asteraceae), *Wolfiporia extensa* (Peck) Ginns (Polyporaceae), *Glycyrrhiza uralensis* Fisch ex DC. (Fabaceae), *Zingiber officinale* Rosc. (Zingiberaceae), and *Mentha canadensis* L. (Lamiaceae) (Tian et al. 2016; Liver Disease Committee 2019). Our previous studies have shown that XYS treatment alleviates liver injury and improves liver fibrosis *in vivo* and *in vitro* through the TGF-β/Smad and Akt/FoxO3 signalling pathways (Zhou et al. 2021). In addition, the formula also exerts anxiolytic-like effects through downregulation of TNF-α/JAK2-STAT3 signalling (Li et al. 2017). Due to the complexity of CHFs from their multiple compounds, targets, and biological activities, it has been difficult to assess their real and multiple compounds combined pharmacological effects and mechanisms. The recent development of transcriptomics, network pharmacology and systems biology provide novel methodologies for such analyses and a better understanding of the molecular mechanism for different CHFs, such as Huangqi decoction (Song et al. 2016), Yinchenhao decoction (Cai et al. 2019), and Fuzhenghuayu formula (Dong et al. 2018). These results also provide approaches for determining the synergistic effects of active compounds from XYS on liver fibrosis.

In this study, the mRNA of chronic hepatitis B patients with LDSDS was analyzed to clarify the specific genes that XYS targeted. Combined with TCM databases, we developed a novel formula with luteolin, licochalcone A, aloe-emodin, and acacetin (LLAAF) from XYS based on pharmacology network analysis, predicted the anti-liver fibrotic mechanisms using transcriptomics analysis, and verified their anti-liver fibrotic mechanisms *in vitro* and *in vivo*.

## Materials and methods

### Reagents

Luteolin, licochalcone A, aloe-emodin and acacetin (all ≥98% pure by HPLC) were purchased from Shanghai Traditional Chinese Medicine Standardization Centre (Shanghai, China). CCl_4_ and olive oil were obtained from Sinopharm Chemical Reagent Co., Ltd. (Shanghai, China). Alanine aminotransferase, aspartate aminotransferase, albumin, and total bilirubin kits were purchased from Shino-Test Corporation (Tokyo, Japan). The hyaluronic acid, laminin, type IV collagen, and type III procollagen kits were from Shanghai Enzyme Biotechnology Co., Ltd. (Shanghai, China). Hydroxyproline (Hyp) detection kit was purchased from Nanjing Institute of Bioengineering (Nanjing, China), and colchicine was from Banna Pharmaceutical Co., Ltd. (Xishuangbanna, China). Methanol (≥98% pure by HPLC) and formic acid (≥98% pure by HPLC) were from Thermo Fisher Scientific (Waltham, MA, USA). The antibodies against human smooth muscle actin (α-SMA) and type I collagen A2 (collagen IA2) were obtained from Abcam (Cambridge, MA, USA); the antibodies against p-Akt, Akt, p-Smad3, Smad3, p-STAT3, p-FoxO3a, and GAPDH were from Cell Signalling Technology Inc. (Danvers, MA, USA); and the antibodies against c-Myc, FoxO3a, and STAT3 were obtained from Proteintech (Rosemont, IL, USA). Foetal bovine serum (FBS) and Dulbecco’s modified Eagle’s medium (DMEM) were obtained from Gibco Life Technologies (Gaithersburg, MD, USA), and human TGF-β1 was from Promega (Madison, WI, USA).

### Preparation of XYS

Eight Chinese herbals comprising XYS were purchased from Shanghai Huanghai Pharmaceutical Co., Ltd. (Shanghai, China), and prepared following established methods (Zhou et al. 2021), which contained 10 g of *Bupleurum chinense*, 10 g of *Angelica sinensis*, 10 g of *Paeonia lactiflora*, 10 g of *Atractylodes macrocephala*, 10 g of *Wolfiporia extensa*, 5 g of *Glycyrrhiza uralensis*, 3 g of *Zingiber officinale*, and 3 g of *Mentha canadensis*. The medicinal herb mixture was soaked in 2 L of water for 30 min, boiled for 30 min to obtain aqueous extracts, then the extraction was filtered and concentrated to 0.63 g crude drug/mL as XYS decoction for the experiments and stored at −20 °C before use.

### Analysis of XYS active compounds and potential gene targets

The active compounds and potential targets of XYS were retrieved from five different TCM databases (TCM@Taiwan, TCMSP, TCMID, TCM-PTD, and HIT). We selected potential active compounds of XYS according to their pharmacokinetic and pharmacodynamic properties (oral bioavailability, OB ≥ 30% and drug-likeness, DL ≥ 0.18). Next, we identified the potential target proteins of XYS through the molecular similarity match tool, SMILES in SEA (http://sea.bkslab.org/) and Swiss for target prediction. The liver fibrosis-related targets using the three human diseases databases, including OMIM, Genecards, and Disgene, with the keywords ‘hepatic fibrosis’ and ‘liver fibrosis’. After that, we expanded the target protein prediction from the STRING database according to the human protein-protein interactions (PPIs).

### mRNA microarray analysis

Blood samples from chronic hepatitis B patients with or without LDSDS (n = 16 for each group) were collected. This study was approved by the Ethics Committee of Shanghai University of Traditional Chinese Medicine, and all participants provided a written informed consent form before enrolment. Cellular RNA from blood cells were purified using RNeasy Mini Kit (QIAGEN, Hilden, Germany) according to the manufacturer’s protocol. The RNA integrity number was determined by Agilent Bioanalyzer 2100 (Agilent Technologies, Santa Clara, CA, USA), and then stored at −80 °C until use. For the cDNA microarray analysis, samples were labelled using Low Input Quick Amp Labelling Kit, and hybridized to the cDNA array by Gene Expression Hybridization Kit (Agilent Technologies). The arrays were scanned by Agilent Microarray Scanner (Agilent Technologies) with default settings, and the raw data were analyzed by the Feature Extraction software 10.7 (Agilent Technologies) and normalized using the Quantile algorithm and Gene Spring Software 11.0 (Agilent Technologies). The transcriptomic data of the differentially expressed genes (DEGs) were obtained after the SAS online software analysis for a fold change >1.5 and *p* < 0.05.

Similarly, liver tissues from the CCl_4_-treated control and XYS or LLAAF treated rats (*n* = 3 for each group) were collected for total cellular RNA isolation and cDNA microarray analysis. For the criteria of DEGs, we utilised a fold change >2 and *p* < 0.05 as the criteria.

### Bioinformatics analyses of the DEGs

We constructed XYS-induced DEG networks and XYS- or LLAAF-targeted mRNA networks using Cytoscape software and calculated the related parameters for significant network nodes according to a previous study (Shi et al. 2014). We evaluated the network stabilization according to the method of Liu et al. (2017). We then utilized the GeneMAIA tool to construct the PPI network of LLAAF and its four individual compounds and the MCODE tool in Cytoscape software to extract their core subnetwork using *p* < 0.05. After that, we compared the composition of the core subnetwork using the Venn analysis tool and the Omicsbean tool plus the Venn analysis tool to predict the mechanism of action of these different compounds. In addition, we performed gene ontology (GO) and Kyoto Encyclopaedia of Genes and Genomes (KEGG) database pathway enrichment analyses using the online Omicsbean analysis tool.

### Prediction of potential compound combinations of XYS

We evaluated the relevant network stability parameters, including the network centralization (NC), characteristic path length (CPL), network heterogeneity (NH) and robustness (R) levels using Cytoscape software, according to a previous study (Liu et al. 2017). The R levels were calculated using the following formula:

$$R=\frac{C}{\left(N\;-\;Nr\right)}$$

where *C* is the maximum connected amount, *N* is the number of original nodes, and *Nr* is the number of knockout nodes.

We then calculated the network contribution score and the importance of the compound in the network using the entropy method and the weighted summation method. Next, we performed a Venn analysis to retain the top 50% of the screening scores. The network contribution scores were calculated using the following methodologies by converting the actual value of each relevant network stability parameter into an evaluation value:

$$\text{Positive indicator}:\;\mathrm{zij}=\frac{\left.\mathrm{xij}-\min(xj\right)}{\left.\max(xj)-\min(xj\right)}$$

$$\text{Inverse indicator}:\;\mathrm{zij}=\frac{\max(xj)-\mathrm{xij}}{\left.\max(xj)-\min(xj\right)}$$

$$\text{Weight}\;\text{of}\;\text{each}\;\text{indicator:}\;\mathrm{bij}=\frac{\mathrm{zij}}{\sum ni=1zij},i=1,\ldots ,n,j=1,\ldots ,m$$

$$\text{Entropy value of each indicator}:\;\left.ej=-k\sum_{i=1}^{n}\mathrm{bjln}(bj\right);k=1/ln(n);\;k>0;\;ej>0$$

Difference coefficient: dj=1−ej

The difference coefficient was normalized to obtain the weight of each index:

$$wj=\frac{dj}{\sum_{i=1}^{n}dj}$$

The formula for the network contribution score is as follows:

$$\left.\text{Contribution score }=NC\times W(\text{nc})j+\;\mathrm{CPL}\times W(\text{cpl})j+\;NH\times W(NH)j+R\times (W(R)j\times 2\right)$$

The high-scoring compounds and combinations were then selected for subsequent experimental verification.

### Animal experiments

The animal protocol of this study was approved by the Institutional Animal Care and Use Committee of Shanghai University of Traditional Chinese Medicine (Shanghai, China) and followed the Guidelines for the Care and Use of Laboratory Animals issued by the Chinese Council on Animal Research. Specifically, male Wistar rats weighing 150–160 g were purchased from the Shanghai Laboratory Animal Centre of the Chinese Academy of Sciences (Shanghai, China) and acclimated to the laboratory conditions for 7 days at room temperature (22–26 °C) with a relative humidity of 40–70%. The rats were randomly allocated to the control (*n* = 10) or the CCl_4_ treatment group (*n* = 30). The rats were intraperitoneally injected with 1 mL/kg bodyweight of olive oil or 50% CCl_4_ in olive oil at 1 mL/kg bodyweight, twice a week for 9 weeks. At the end of week 4, the CCl_4_-treated rats were then randomly divided into three groups: the control model group (*n* = 10), the XYS treatment group (*n* = 10), and the LLAAF treatment group (*n* = 10). These rats were treated orally with normal saline, XYS extract (method of decocting) at a dose of 6.3 g/kg/d, and LLAAF (5.5 mg/kg/d luteolin, 1.5 mg/kg/d licochalcone A, 2.5 mg/kg/d aloe-emodin, and 2.5 mg/kg/d acacetin) daily for 4 weeks, respectively. If the animal weight loss reached 20–30% of that of the control rats, the model rats were euthanized by CO_2_ and then cervical dislocation. At the end of the experiments, all rats were sacrificed for tissue resection and various analyses.

### Histopathological analysis of rat liver tissues

Rat liver tissues were collected, weighed, and sectioned into small pieces. Then, they were fixed in 4% paraformaldehyde in phosphate buffer and subjected to paraffin embedding for haematoxylin and eosin (H&E) staining and Masson’s staining, according to a previous study (Dong et al. 2018). The Masson-stained tissue sections were quantified by ImageJ software (National Institutes of Health, Bethesda, MD, USA).

### Hepatic Hyp content analysis

Liver tissue homogenates were sonicated for complete dissolution and centrifuged at 12,000 rpm for 30 min at 4 °C to separate the membrane-containing fraction (pellet) from the cytosolic fraction. Hyp in liver tissues were measured according to the instructions provided by the manufacturers of the corresponding analytical kits.

### Cell culture and treatment

The human HSC lines LX2 and LO2 as well as the rat HSC cell line HSC-T6, were obtained from the Type Culture Collection of the Chinese Academy of Sciences (Shanghai, China), and maintained in DMEM supplemented with 10% FBS, penicillin (100 U/mL), and streptomycin (100 μg/mL) in a humidified incubator with 5% CO_2_ at 37 °C, according to a previous study (Song et al. 2016). For cell treatment, HSC-T6 and LX2 cells were cultured in a 6-well plate at a density of 1.0 × 10^6^ cells/well overnight, and then they were treated with TGF-β1 (LX2, 5 ng/mL and HCS-T6, 20 ng/mL) and compounds or their combinations, that is, the compound concentrations were 0.8–3.1 μM luteolin, 0.8–3.1 μM licochalcone A, 3.1–12.5 μM aloe-emodin, and 1.5–6.3 μM acacetin, for 24 h.

### Cell viability assay

Cells were seeded into 96-well plates at a density of 5 × 10^3^ cells/well, grown overnight, and then treated with the above-named compounds and their combinations for 24 h. At the end of each experiment, 20 μL of the tetrazolium compound 3-(4,5-dimethylthiazol-2-yl)-5-(3-carboxymethoxyphenyl)-2-(4-sulfophenyl)-2H-tetrazolium (MTT) (Thermo, Waltham, MA USA) was added to the cell culture, the cells were incubated for an additional 3.5 h, and the absorbance rates were measured using a Synergy2 spectrophotometer (BioTek, Winooski, VT, USA) at 490 nm. The experiment was performed in triplicate and repeated at least three times.

### Western blot

Western blot procedure was followed by the previous study (Zhou et al. 2021). In detail, the Protein samples from liver tissue or cells of each were separated by 10% SDS-polyacrylamide gel electrophoresis and transferred onto nitrocellulose membranes (Millipore, Billerica, MA, USA). The membrane was incubated with a blocking buffer containing 5% non-fat dry milk in Tris-based saline-Tween 20 (TBS-T) at room temperature for 1 h and then with polyclonal primary antibodies at 4 °C overnight. On the next day, the membranes were washed with TBS-T three times for 10 min each and then incubated with secondary antibody at room temperature for 1 h. The protein bands were quantified using an Odyssey Infrared Imager (LI-COR Biosciences, Lincoln, NE, USA), according to a previous study (Liu et al. 2017).

### Statistical analysis

The data were expressed as the mean ± standard deviation for the one-way analysis of variance and rank-sum tests using SPSS 18.0 software (SPSS, Chicago, IL, USA). A *p-*value <0.05 was considered statistically significant.

## Results

### Identification of active compounds, targets and signalling pathways of XYS

**Figure 1.**
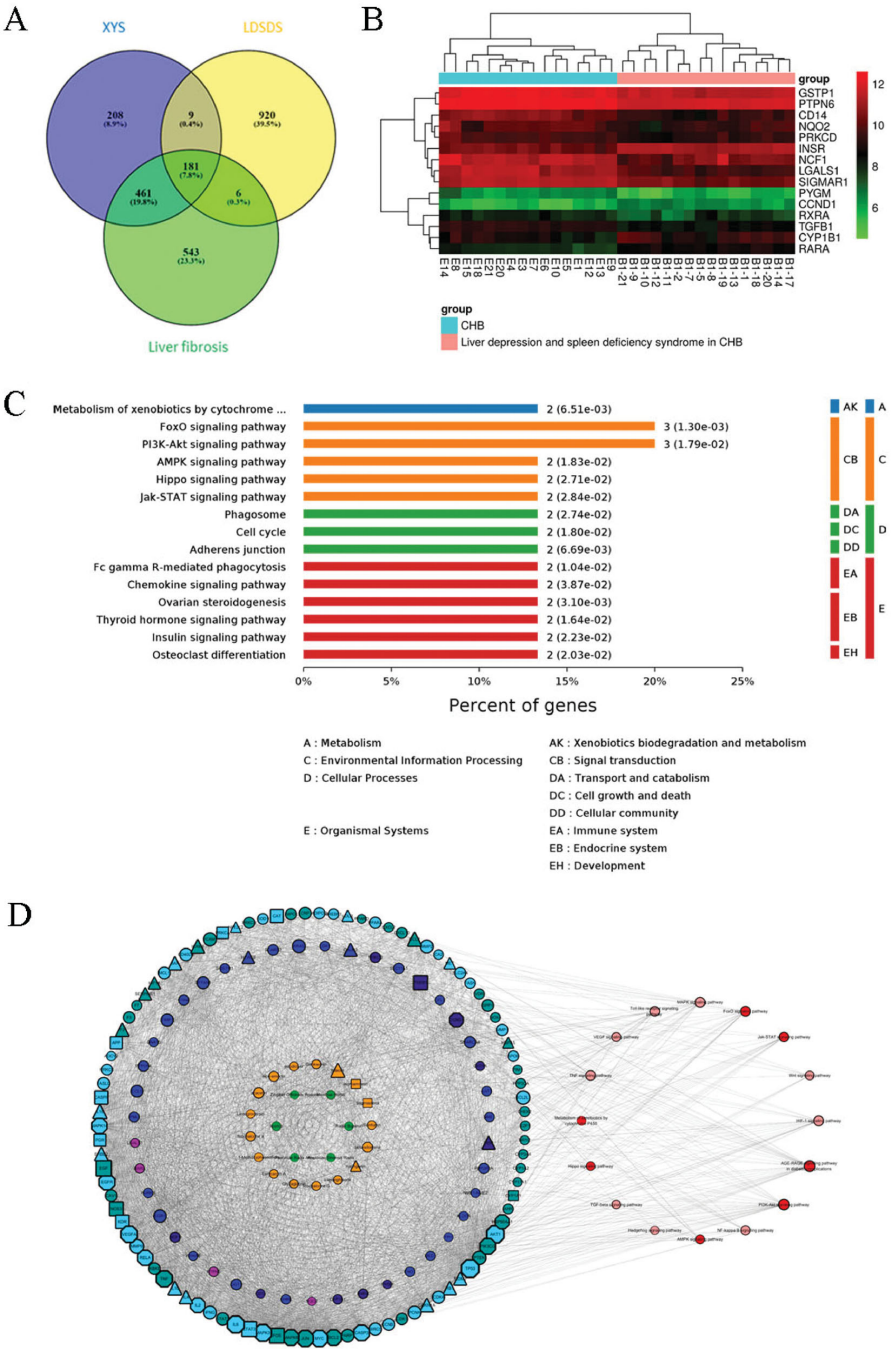
Identification of the active compounds, gene targets, and signalling pathways of XYS. (A) Venn analysis of differentially expressed genes (DEGs) in the XYS, LDSDS, and liver fibrosis groups. (B) Heatmap of DEGs between chronic hepatitis B patients with or without LDSDS (*n* = 16 in each group). (C) The KEGG pathway analysis directly related to XYS. (D) The XYS-LDSDS-liver fibrosis core network analysis. Green, herbs; orange, compounds; indigo, target proteins directly related to XYS; purple, predicted target proteins directly related to XYS; dark blue, target proteins indirectly related to XYS; light blue, target proteins intermediately related to XYS; dark green, predicted target proteins intermediately related to XYS; deep red, core pathway; pink, noncore pathway.

In this study, we first screened and found a total of 174 active compounds and 258 potential target proteins in XYS from the five TCM databases using our pharmacokinetics and pharmacodynamics screening criteria (OB ≥ 30% and DL ≥ 0.18). After performing the SwissDock and SEA structural similarity prediction analyses, 525 predicted target proteins were expanded. A total of 601 related target proteins were found in the PPI network analysis (PPI score >0.850) from 258 primary target proteins. In total, 859 XYS-effect related targets were found. In addition, 1191 liver fibrosis-related targets were found by the OMIM, DisgeneT, and GeneCard databases using the keywords ‘hepatic fibrosis’ and ‘liver fibrosis’. In LDSDS patients, 1116 DEGs were identified from the mRNA microarray data. When comparing the DEGs of the XYS, LDSDS, and liver fibrosis groups, 181 targets were identified for the XYS-LDSDS-liver fibrosis network (Figure 1(A)). Compared with the XYS active compound-related target proteins, the data showed 14 target proteins directly related to XYS. The KEGG pathway enrichment analyses showed that these 14 XYS target genes were focussed on the PI3K-Akt-FoxO, AMPK, Hippo, and Jak-STAT pathways (Figure 1(C)). The heatmap of these targets between chronic hepatitis B patients with or without LDSDS is shown in Figure 1(B).

Next, the network for the XYS-LDSDS-liver fibrosis and active compounds was constructed. The nodes of the compound-target network included 165 compounds, 14 proteins directly targeted by XYS from the TCM database, 166 proteins indirectly targeted by XYS from structural similarity prediction, and 184 intermediately targeted proteins from the PPI network. The core network of XYS-LDSDS-liver fibrosis progression was obtained using the five most important topological parameters, including degree, betweenness of centrality, the closeness of centrality, clustering coefficient, and topological coefficient analysis. The nodes of the PPI network included six Chinese herbal medicines and 17 compounds, and XYS was directly associated with nine target proteins; XYS was predicted to target 6 proteins directly, 30 indirectly, and 102 intermediately as well as 15 signalling pathways (Figure 1(D)).

### Evaluation of compounds in the XYS-LDSDS-liver fibrosis core network

**Figure 2.**
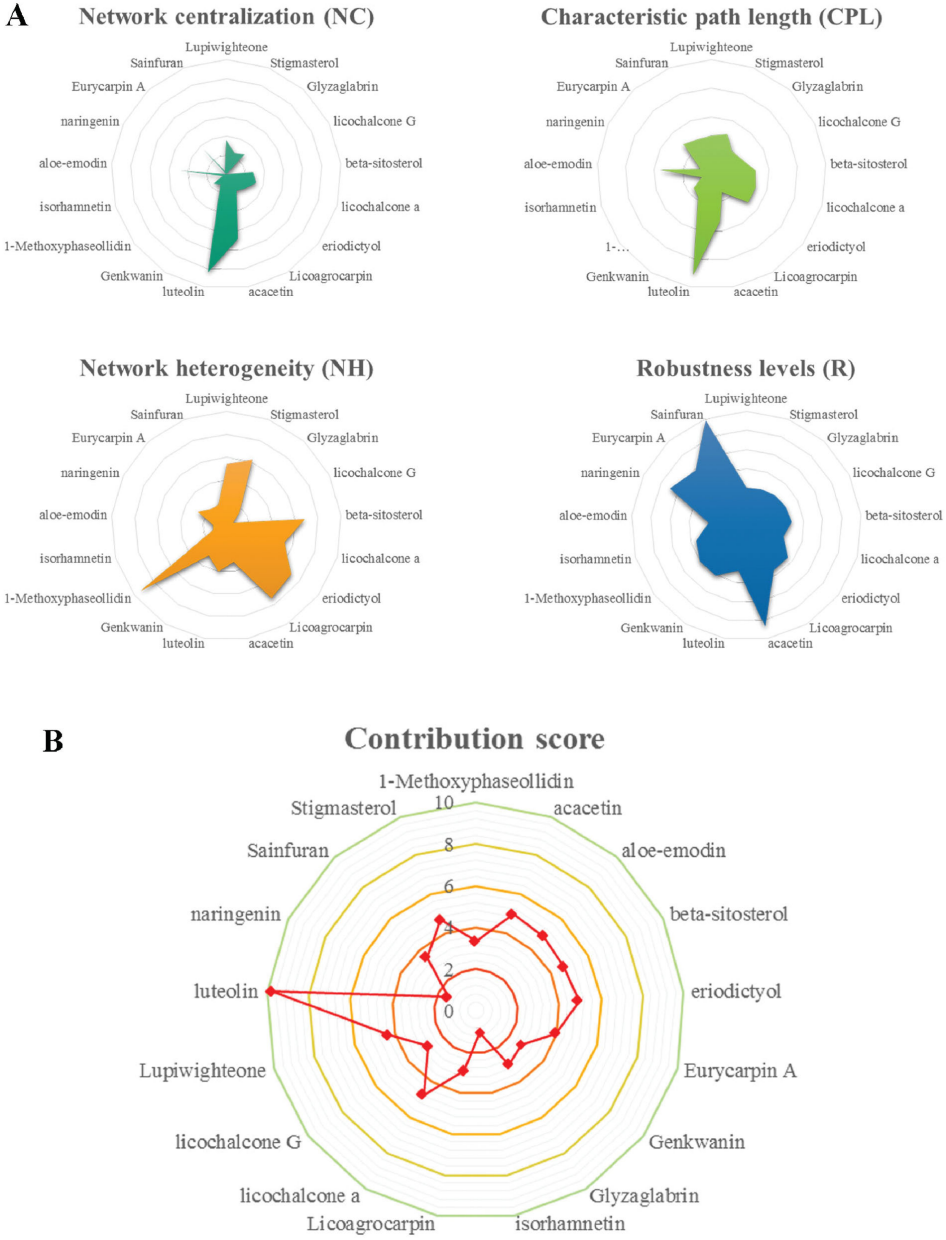
Contribution score analysis of the XYS-LDSDS-liver fibrosis core network. (A) Network centralisation (NC), characteristic path length (CPL), network heterogeneity (NH), and robustness (R) scores of the 17 compounds by using the entropy and the weighted summation methods, which were directly related to the network stability using Cytoscape 3.2.1 software. (B) Analysis of the contribution scores of the 17 compounds by combinations of the NC, CPL, NH and R parameters.

To identify the core compounds of the XYS-LDSDS-liver fibrosis network, we knocked out the related target proteins of 17 compounds and utilized the entropy method as well as the weighted summation method to generate the network contribution scores by integrating the NC, CPL, NH, and R parameters, which are directly related to the stability of the networks, using Cytoscape 3.2.1 software (Figure 2(A)). Consider the contribution score of these four parameters, eight compounds, luteolin, lupiwighteone, beta-sitosterol, stigmasterol, licochalcone A, aloe-emodin, eriodictyol, and acacetin were selected from the 17 compounds. And these eight compounds were made of 255 different combinations. Based on the network contribution score analysis, the top 10 compound combinations and their scores are listed (Table 1).

**Table 1.** Contribution scores of the compound combinations.

Compound combination	Contribution score
Luteolin + β-sitosterol + licochalcone A + aloe-emodin + eriodictyol	18.52698678
Luteolin + licochalcone A + aloe-emodin + eriodictyol	18.07387444
Luteolin + licochalcone A + aloe-emodin + acacetin	17.47842125
Luteolin + β-sitosterol + licochalcone A + aloe-emodin + acacetin	17.44939433
Luteolin + lupiwighteone + stigmasterol + licochalcone A + aloe-emodin + eriodictyol + acacetin	16.90428988
Luteolin + lupiwighteone + β-sitosterol + aloe-emodin + eriodictyol	16.42871462
Luteolin + lupiwighteone + aloe-emodin + eriodictyol	16.12207079
Luteolin + lupiwighteone + β-sitosterol + licochalcone A + aloe-emodin + eriodictyol	15.9687878
Luteolin + lupiwighteone + β-sitosterol + stigmasterol + licochalcone A + aloe-emodin	15.77850732
Luteolin + lupiwighteone + β-sitosterol + aloe-emodin + acacetin	15.37578559

### Effects of XYS compounds and their combinations on regulating cell viability *in vitro*

**Figure 3.**
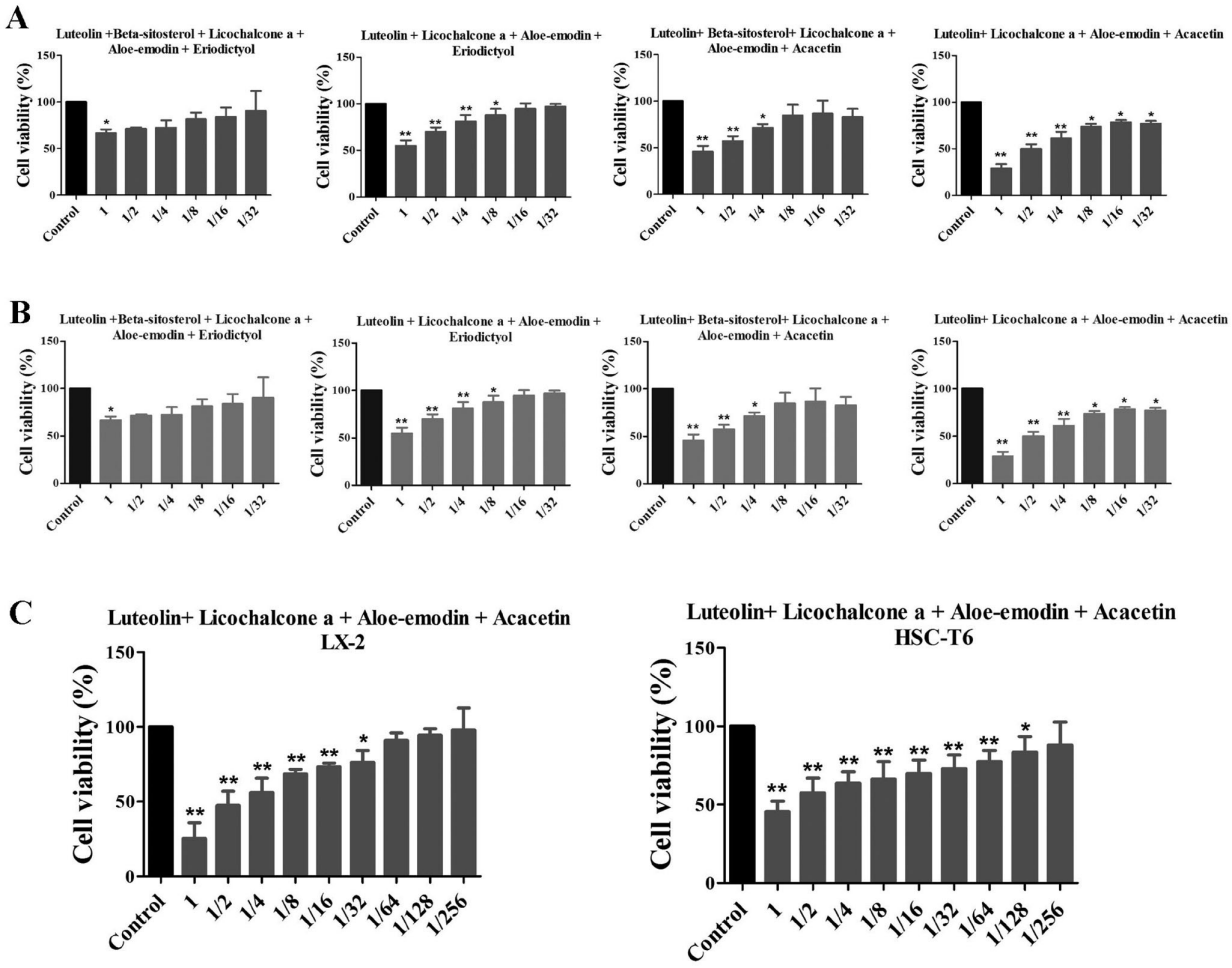
Effects of six individual XYS compounds and their combinations on regulating cell viability. (A) Cell viability assay. The four top compound combinations from network analysis show the effect on the LX2 cells. (B) Cell viability assay. The four top compound combinations from network analysis show the effect on the HSC-T6 cells. *p < 0.05 and **p < 0.01, compared to the control cells. (C) The most active compound combination LLAAF effect on the LX2 and HSC-T6 cells viability.

Since the hepatic stellate cells were important in the liver fibrosis process, we also detect the human HSC viability by compounds treatment. The six single compound’s effects on the cell viability of LX2 cells were detected, and the minimum effective dose of these six individual compounds combined 4 different combinations, also detected the cell viability of LX2 cells (Figure 3(A)). And it was shown that the best effective combination was the luteolin, licochalcone, aloe-emodin, and acacetin formula (LLAAF, Figure 3(C)). Similar findings were confirmed in rat HSC-T6 cells (Figure 3(B,C)).

### Effect of LLAAF on α-SMA and collagen I expression *in vitro*

**Figure 4.**
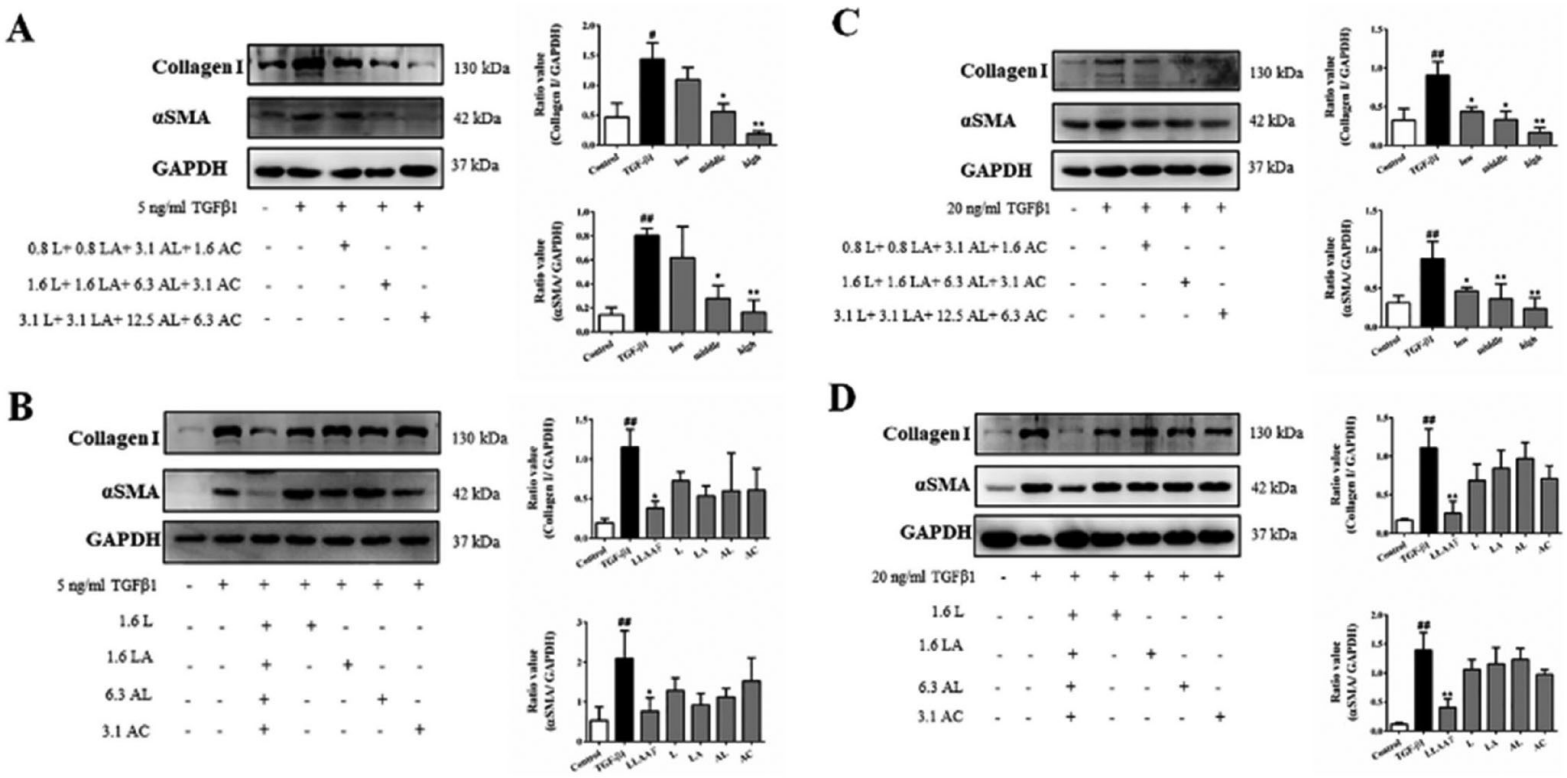
Effect of LLAAF on regulating protein expression *in vitro*. (A) Western blot analysis of α-SMA and collagen I expression in LX2 cells with treated with a high, medium, or low dose of LLAAF. Quantified data of the western blots are shown in the corresponding graphs. (B) Western blot analysis of α-SMA and collagen I expression in LX2 cells with treated with a medium dose of LLAAF or its individual compounds. (C) Western blot analysis of α-SMA and collagen I expression in HSC-T6 cells with treated with a high, medium, or low dose of LLAAF. Quantified data of the western blots are shown in the corresponding graphs. (D) Western blot analysis of α-SMA and collagen I expression in HSC-T6 cells with treated with a medium dose of LLAAF or its individual compounds. Quantified data of the western blots are shown in the corresponding graphs. **#***p* < 0.05 and **##***p* < 0.01, compared to the control cells; *p < 0.05 and **p < 0.01, compared to the TGFβ group.

αSMA and collagen I expression can reflect liver fibrosis content. Based on the effective doses of LLAAF on reducing the LX2 cell viability, 3 doses were used to detect αSMA and collagen I expression as 3.1 μM luteolin + 3.1 μM licochalcone A + 12.5 μM aloe-emodin + 6.3 μM acacetin; 1.6 μM luteolin + 1.6 μM licochalcone A + 6.3 μM aloe-emodin + 3.1 μM acacetin; 0.8 μM luteolin + 0.8 μM licochalcone A + 3.1 μM aloe-emodin + 1.6 μM acacetin. The levels of α-SMA and collagen I were significantly increased in LX2 and HSC-T6 cells by TGF-β1. And the high and medium doses of LLAAF were able to dramatically reduce the expression of α-SMA and collagen I in these cells (*p* < 0.05), but the low-dose LLAAF treatment only reduced the expression levels of α-SMA and collagen I in HSC-T6 cells (*p* < 0.05; Figure 4(A,C)). However, any single compound at the same concentration did not show any effects on changing α-SMA and collagen I expression in these cells (Figure 4(B,D).

### Effects of XYS and LLAAF on liver fibrosis *in vivo*

**Figure 5.**
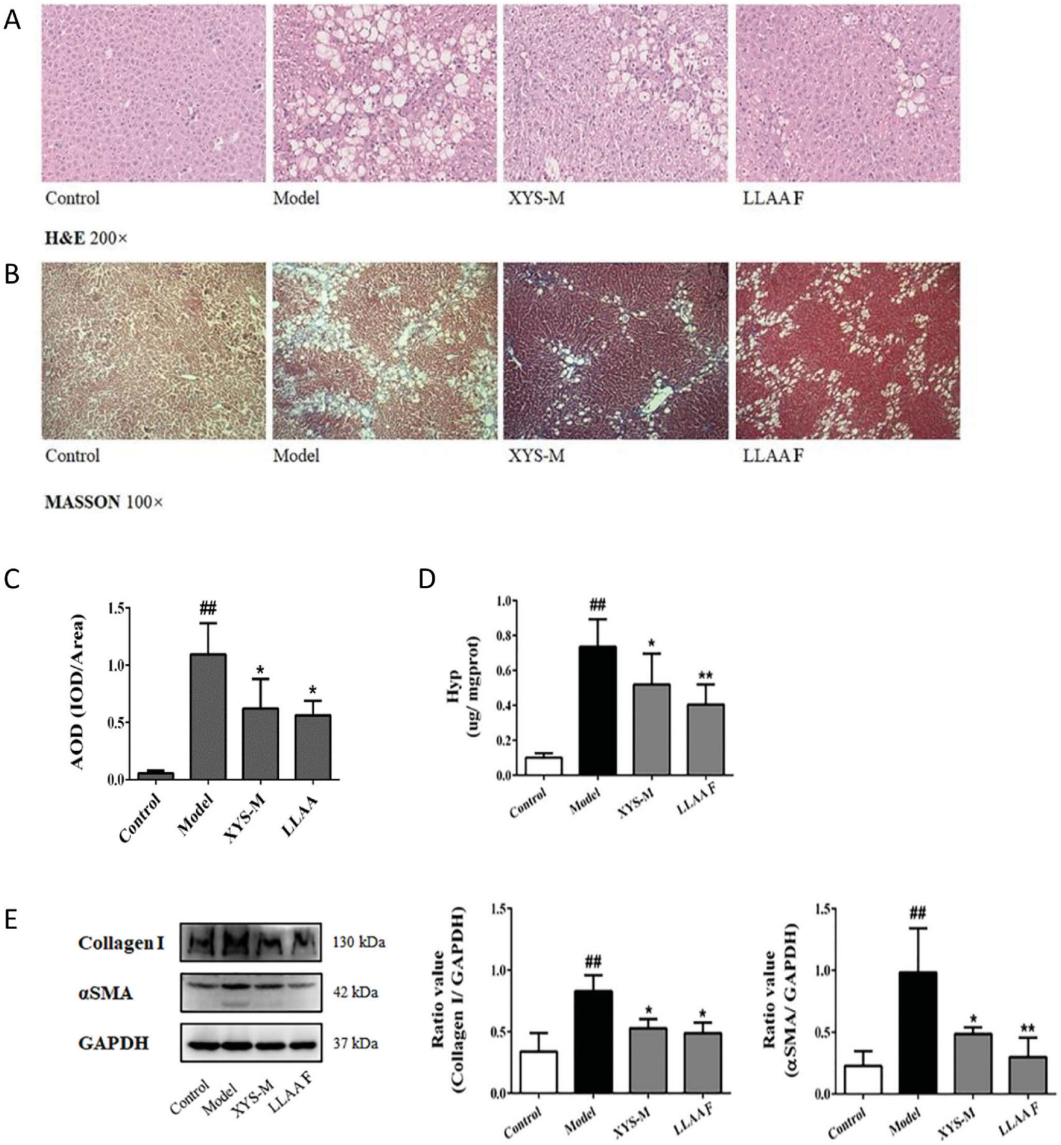
Effects of XYS-M and LLAAF treatment on the reduction in rat liver fibrosis. (A) H&E staining (200× magnification). (B) Masson’s staining (100×). (C) Semi-quantitative analysis of the collagen fibres. (D) The hydroxyproline (Hyp) content. (E) The levels of alpha-smooth muscle actin (α-SMA) and collagen I were analysed using western blot of rat liver fibrotic tissues. **##***p* < 0.01, compared to the control group; *p < 0.05, **p < 0.01, compared to the model group.

The effects of LLAAF and XYS were evaluated in the CCl_4_ induced liver fibrosis model of Wistar rats. In a previous study, XYS was able to improve the rat liver weight index, pathological index, serum liver function index, serum liver fibre index, and liver tissue fibres *in vivo* (Zhou et al. 2021). The histopathological data showed the establishment of CCl_4_-induced liver fibrosis in rats treated twice a week for 9 weeks (Figure 5), whereas treatment of rats with either LLAAF or XYS significantly attenuated the CCl_4_-induced liver fibrosis. Specifically, H&E staining showed lower levels of oedema and inflammation of liver cells in the XYS and LLAAF groups than in the CCl_4_ controls (Figure 5(A)), whereas Masson’s staining showed that fibrosis and paraplastic connective tissues were built up in the CCl_4_ rats vs. mild fibrosis in the XYS and LLAAF rats (Figure 5(B)). This semi-quantitative analysis indicated reduced levels of collagen fibres in the XYS and LLAAF-treated rats compared to the model rats (*p* < 0.01; Figure 5(C)). Furthermore, the Hyp content in the XYS and LLAAF-treated rats were decreased compared with the CCl_4_-induced rat liver tissue (*p* < 0.05; Figure 5(D)). And western blot analysis also revealed that the levels of α-SMA and collagen I were reduced significantly (*p* < 0.05 or *p* < 0.01; Figure 5(D)). In addition, treatment with LLAAF showed an even better reduction in the Hyp content and α-SMA level in the liver tissues.

### Prediction of the anti-liver fibrotic mechanisms of XYS and LLAAF

**Figure 6.**
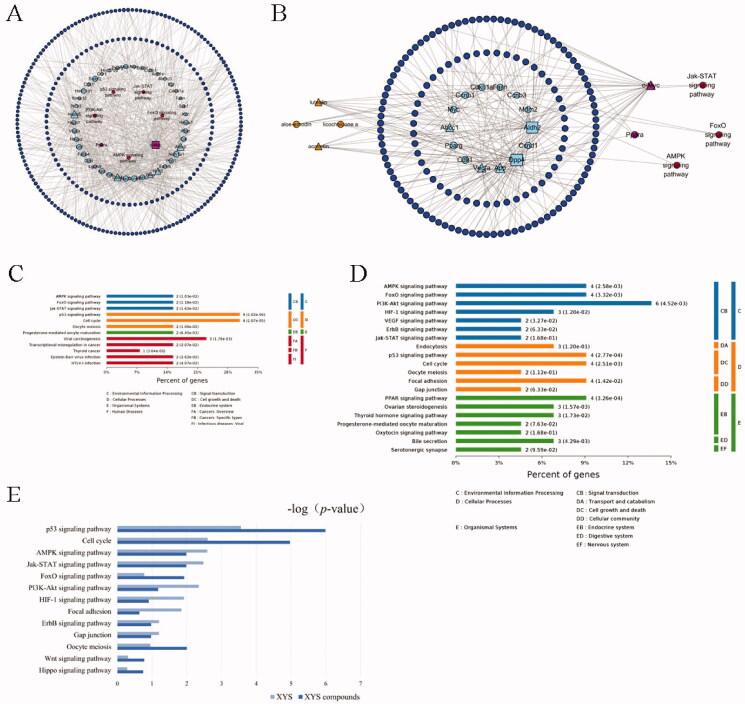
Bioinformatics analysis of the XYS- and LLAAF-modified PPI networks. (A) XYS network. (B) LLAAF network. (C) KEGG enrichment analysis of the XYS network. (D) KEGG enrichment analysis of the LLAAF network. (E) KEGG Pathway analysis compared with XYS and LLAAF.

To explore the underlying mechanisms of the anti-fibrotic efficacy of XYS and LLAAF, we collected liver tissue samples from the CCl_4_ model rats, XYS-treated rats, and LLAAF-treated rats for cDNA and lncRNA microarray analyses. And the total DEGs of XYS and LLAAF compared with the CCl_4_ model group were analyzed (Figure 6(A,B)). The corresponding KEGG pathways were also analyzed individually. (Figure 6(C,D)) When comparing the two KEGG pathways, XYS treatment was mainly related to p53, cell cycle, viral carcinogenesis, Jak-STAT3, AMPK, and FoxO signalling, while the LLAAF treatment was mainly associated with PI3K-Akt, AMPK, FoxO, Jak-STAT3, P53, cell cycle, focal adhesion, and PPAR signalling (Figure 6(E)).

### LLAAF regulates Jak-STAT and PI3K-Akt-FoxO signalling

**Figure 7.**
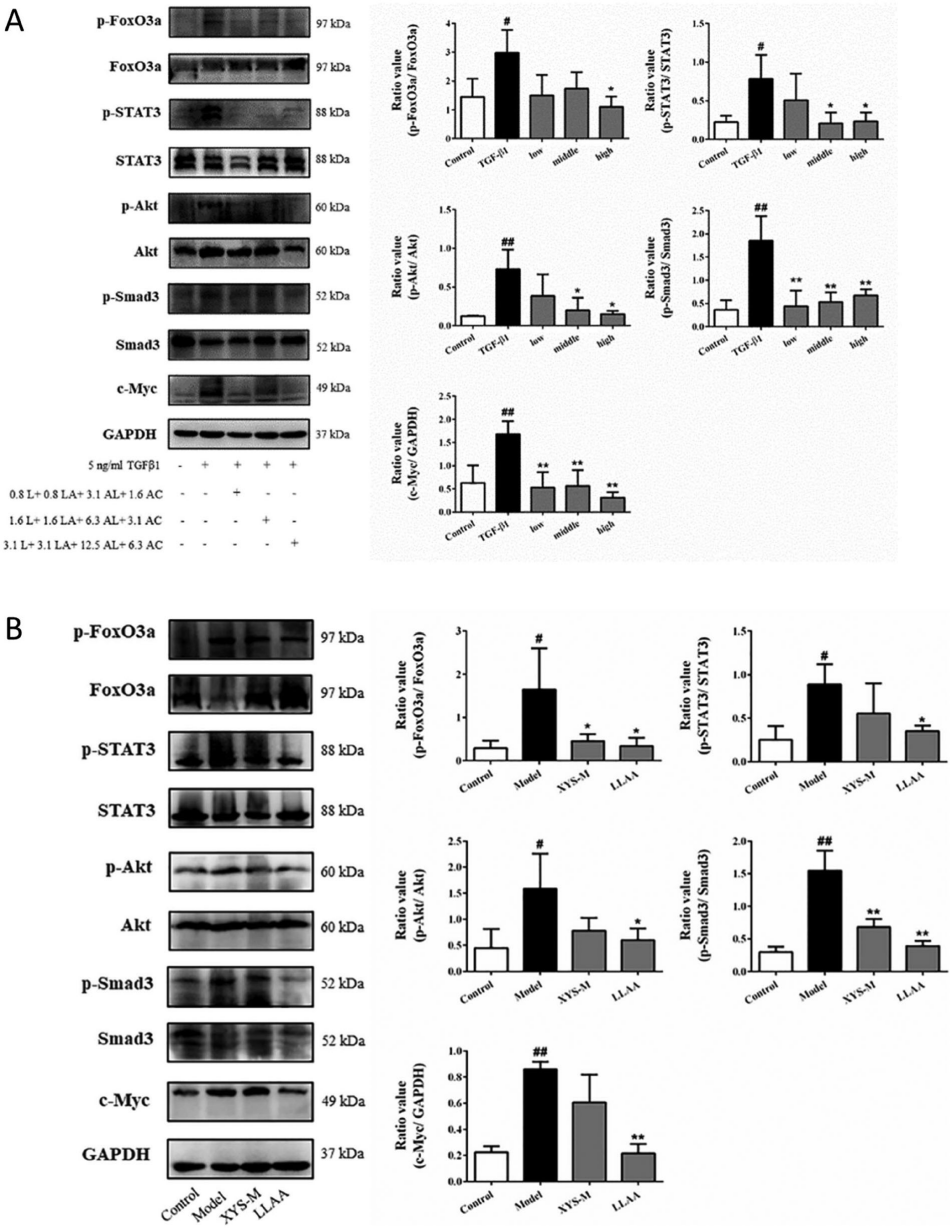
Effect of LLAAF treatment on the regulation of Jak-STAT and PI3K-Akt-FoxO signalling. (A) Western blot analysis of protein expression in LX2 cells were grown, treated with TGF-β1 and LLAAF. (B) Western blot analysis of protein expression in liver tissues. Quantified data of the western blots are shown in the corresponding graphs. #*p* < 0.05 and ##*p* < 0.01, compared to the cell control or rat control group; *p < 0.05 and **p < 0.01, compared to TGF-β1 or the CCl_4_ model group.

These bioinformatics analysis results were performed *in vitro* and *in vivo*. The LX2 cells were treated with TGF-β1, an inducer of liver fibrosis. The treatment significantly increased the levels of p-FoxO3a, p-STAT3, p-Akt, p-Smad3, and c-Myc in LX2 cells (*p* < 0.05; Figure 7(A)); whereas different doses of LLAAF treatment significantly downregulated their expression (*p* < 0.05; Figure 7(A)). We also detected the Jak-STAT and PI3K-Akt-FoxO signalling in the liver samples from an animal model, and found that LLAAF significantly downregulated the Jak-STAT and PI3K-Akt-FoxO signalling similar to the XYS (Figure 7(B)).

## Discussion

Liver fibrosis is a liver wound healing process that responds to different types of liver disease-induced hepatocyte injury. It reflects an imbalance between fibrosis progression and regression (Yoon et al. 2016) and confers a significant risk for morbidity and mortality (Hernandez-Gea and Friedman 2011). The advanced stage of liver cirrhosis is only cured by liver transplantation (Koyama and Brenner 2015); thus, novel treatment options and a better understanding of the underlying pathogenesis are critical to cure and improve the quality of life of these patients. In a previous study, we evaluated a Chinese herbal formula named XYS, which has anti-liver fibrotic activity *in vitro* and *in vivo* (Zhou et al. 2021). Network pharmacology combined with transcriptomics analysis was used to identify active compounds and gene targets of XYS. 174 active compounds in XYS and participation of the PI3K-Akt-FoxO, AMPK, Hippo and Jak-STAT pathways. Indeed, XYS contains more than 174 active compounds, making it difficult to assess those responsible for controlling liver fibrosis *in vivo* and *in vivo*.

In the current study, by analyzing the topology parameters according to previous studies (Shi et al. 2014; Wieczorek et al. 2015; Han et al. 2017), we constructed the XYS-LDSDS-liver fibrosis core networks, based on the network contribution score analysis for their feasibility ranking. We utilized four parameters, that is, NC, CPL, NH, and R, to relate the stability of the bioinformatics networks according to a previous study (Eloundou-Mbebi et al. 2016). The method of entropy weighting is a common way to integrate multiple indicators (Harvey et al. 2017); the greater the entropy of an indicator is, the greater the impact of the indicator on the comprehensive evaluation. After evaluating the network contribution scores, we identified eight core compounds and the top 10 combinations with anti-fibrotic activities. In the cell-based assay, the combination of luteolin, licochalcone A, aloe-emodin and acacetin was generated as the most effective combination from XYS.

Specifically, luteolin is in a variety of dietary sources, such as celery, broccoli, green pepper, and parsley (Shimoi et al. 1998; Lopez-Lazaro 2009). In addition, luteolin can attenuate the inflammatory responses in dendritic cells, indicating its therapeutic potential against ulcerative colitis (Kim et al. 2019), and protects against CCl_4_-induced hepatotoxicity in combination with metformin (Yan et al. 2019). Moreover, licochalcone A, a chalconoid, can be found in the roots of *Glycyrrhiza glabra* Linn. or *Glycyrrhiza inflata* Batal. (Chen et al. 1994; Friis-Moller et al. 2002; Fu et al. 2004; Dao et al. 2011). It possesses antiproliferative and proapoptotic activities by reactive oxygen species-mediated cell cycle arrest and apoptosis in human bladder cancer cells (Hong et al. 2019) and inhibits proliferation, migration, and invasion of oral squamous cell carcinoma cells by regulating the PI3K/AKT signalling pathway (Hao et al. 2019). Similarly, aloe-emodin, which is isolated from aloe and *Rheum palmatum* Linn., has antiproliferative effects and induces cell apoptosis (Acevedo-Duncan et al. 2004; Guo et al. 2007). It also inhibits monolayer growth and anchorage-independent growth of androgen refractory prostate cancer PC3 cells by targeting the mTOR complex II (Liu et al. 2012). More importantly, aloe-emodin has shown the potential to treat hepatitis B viral infection (Parvez et al. 2019), a condition that induces liver fibrosis, and to alleviate cardiac fibrosis via suppression of cardiac fibroblast activation by metastasis-associated protein 3 upregulation (Xiao et al. 2019). Functionally, acacetin has been reported to regulate the reciprocal differentiation of T helper 17 and regulatory T cells as well as the symptoms of collagen-induced arthritis in mice (Liu et al. 2018). Additionally, it protects cardiomyocytes against hypoxia/reoxygenation injury by activating a series of intracellular signals involved in antioxidation, anti-inflammation, and antiapoptosis (Wu et al. 2018). Taken together, these four compounds all possess anti-inflammatory, antioxidative, antiproliferative, antibacterial, and antiviral activities through multiple gene pathways. Therefore, their combination could yield an even greater anti-liver fibrotic activity *in vitro* and *in vivo*, as demonstrated in the current study.

The anti-fibrotic effect of XYS and LLAAF were classified in cells and in the CCl_4_-induced liver fibrosis rats. HSCs are the most important cell type involved in liver fibrosis and the major producer of the extracellular matrix during liver injury (Eng and Friedman 2000; Geerts 2001). Thus, the current study utilized human and rat HSCs to assess the anti-liver fibrotic activity of the top four compound combinations. These data indicate that the combination of LLAAF was the most effective for cell viability and suppression of α-SMA and collagen I expression *in vitro*. In the CCl_4_-induced liver fibrosis rat model, both XYS and LLAAF were able to reduce cell damage and steatosis, the Hyp content, and the levels of α-SMA and collagen I in liver tissues, suggesting that LLAAF could alleviate liver fibrosis. We performed cDNA microarray analysis and then analyzed the PPI network of the XYS-targeting proteins and the LLAAF-targeting proteins. According to the network topology characteristics and the pathway enrichment analysis, we found that the Jak-STAT and PI3K-Akt-FoxO signalling pathways were the main pathways involved; while previous studies have revealed that Jak-STAT signalling is involved in liver fibrosis and that activation of the Jak-STAT signalling pathway can promote the development and progression of liver fibrosis (Kong et al. 2012; Tang et al. 2017). Other studies have shown that STAT3 phosphorylation occurs during liver fibrosis (Lakner et al. 2010) and that PI3K-Akt-FoxO signalling is involved in chronic liver disease and liver fibrosis (Park et al. 2009). Again, Akt activation leads to FoxO phosphorylation and departure from the cell nucleus, and reduced FoxO expression also results in the development of liver fibrosis. Besides, we also assessed another classic target, Smad3 (Massague 1998), which is highly relevant to liver fibrosis and acts as a signalling mediator for the TGF-β family for cell proliferation, differentiation, and death (Massague 1998). In addition, blockage of Smad3 phosphorylation has been demonstrated to prevent liver fibrosis (Hernandez-Aquino et al. 2017). In this regard, this study confirmed the p-STAT3/STAT3, p-Smad3/Smad3, p-Akt/Akt, and p-FoxO3a/FoxO3a ratios as well as c-Myc expression *in vitro* and *in vivo*. The XYS modified PPI networks were mainly related to p53, cell cycle, viral carcinogenesis, Jak-STAT3, AMPK, and FoxO signalling; while the LLAAF-modified PPI networks were associated with PI3K-Akt, AMPK, FoxO, Jak-STAT3, P53, cell cycle, focal adhesion, and PPAR signalling.

## Conclusions

The current study developed a novel formula, LLAAF with luteolin, licochalcone A, aloe-emodin and acacetin derived from XYS, and demonstrated the anti-fibrotic effects of XYS and LLAAF. The anti-liver fibrotic mechanism of LLAAF may be related to the downregulation of Jak-STAT and PI3K-Akt-FoxO activity, indicating that LLAAF should be further evaluated as a novel therapeutic formula to control liver fibrosis.

## References

[CIT0001] Acevedo-Duncan M, Russell C, Patel S, Patel R. 2004. Aloe-emodin modulates PKC isozymes, inhibits proliferation, and induces apoptosis in U-373MG glioma cells. Int Immunopharmacol. 4(14):1775–1784. 15531293 10.1016/j.intimp.2004.07.012

[CIT0002] Cai FF, Bian YQ, Wu R, Sun Y, Chen XL, Yang MD, Zhang QR, Hu Y, Sun MY, Su SB. 2019. Yinchenhao decoction suppresses rat liver fibrosis involved in an apoptosis regulation mechanism based on network pharmacology and transcriptomic analysis. Biomed Pharmacother. 114:108863–108872. 30991286 10.1016/j.biopha.2019.108863

[CIT0003] Chen M, Theander TG, Christensen SB, Hviid L, Zhai L, Kharazmi A. 1994. Licochalcone A, a new antimalarial agent, inhibits *in vitro* growth of the human malaria parasite *Plasmodium falciparum* and protects mice from *P. yoelii* infection. Antimicrob Agents Chemother. 38(7):1470–1475. 7979274 10.1128/aac.38.7.1470 PMC284578

[CIT0004] Dao TT, Nguyen PH, Lee HS, Kim E, Park J, Lim SI, Oh WK. 2011. Chalcones as novel influenza A (H1N1) neuraminidase inhibitors from *Glycyrrhiza inflata*. Bioorg Med Chem Lett. 21(1):294–298. 21123068 10.1016/j.bmcl.2010.11.016

[CIT0005] Dong S, Cai FF, Chen QL, Song YN, Sun Y, Wei B, Li XY, Hu YY, Liu P, Su SB. 2018. Chinese herbal formula Fuzheng Huayu alleviates CCl_4_-induced liver fibrosis in rats: a transcriptomic and proteomic analysis. Acta Pharmacol Sin. 39(6):930–941. 29094729 10.1038/aps.2017.150 PMC6256277

[CIT0006] Dong S, Su SB. 2014. Advances in mesenchymal stem cells combined with traditional Chinese medicine therapy for liver fibrosis. J Integr Med. 12(3):147–155. 24861835 10.1016/S2095-4964(14)60022-4

[CIT0007] Eloundou-Mbebi JM, Kuken A, Omranian N, Kleessen S, Neigenfind J, Basler G, Nikoloski Z. 2016. A network property necessary for concentration robustness. Nat Commun. 7:13255–13261. 27759015 10.1038/ncomms13255 PMC5075777

[CIT0008] Eng FJ, Friedman SL. 2000. Fibrogenesis I. New insights into hepatic stellate cell activation: the simple becomes complex. Am J Physiol Gastrointest Liver Physiol. 279(1):G7–G11. 10898741 10.1152/ajpgi.2000.279.1.G7

[CIT0009] Friis-Moller A, Chen M, Fuursted K, Christensen SB, Kharazmi A. 2002. *In vitro* antimycobacterial and antilegionella activity of licochalcone A from Chinese licorice roots. Planta Med. 68(5):416–419. 12058317 10.1055/s-2002-32087

[CIT0010] Fu Y, Hsieh TC, Guo J, Kunicki J, Lee MY, Darzynkiewicz Z, Wu JM. 2004. Licochalcone-A, a novel flavonoid isolated from licorice root (*Glycyrrhiza glabra*), causes G2 and late-G1 arrests in androgen-independent PC-3 prostate cancer cells. Biochem Biophys Res Commun. 322(1):263–270. 15313200 10.1016/j.bbrc.2004.07.094

[CIT0011] Geerts A. 2001. History, heterogeneity, developmental biology, and functions of quiescent hepatic stellate cells. Semin Liver Dis. 21(3):311–335. 11586463 10.1055/s-2001-17550

[CIT0012] Guo J, Xiao B, Zhang S, Liu D, Liao Y, Sun Q. 2007. Growth inhibitory effects of gastric cancer cells with an increase in S phase and alkaline phosphatase activity repression by aloe-emodin. Cancer Biol Ther. 6(1):85–88. 17172820 10.4161/cbt.6.1.3553

[CIT0013] Han Y, Li H, Lang Y, Zhao Y, Sun H, Zhang P, Ma X, Han J, Wang Q, Zhou J, et al. 2017. The effects of acute GABA treatment on the functional connectivity and network topology of cortical cultures. Neurochem Res. 42(5):1394–1402. 28290133 10.1007/s11064-017-2190-3

[CIT0014] Hao Y, Zhang C, Sun Y, Xu H. 2019. Licochalcone A inhibits cell proliferation, migration, and invasion through regulating the PI3K/AKT signaling pathway in oral squamous cell carcinoma. Onco Targets Ther. 12:4427–4435. 31239711 10.2147/OTT.S201728 PMC6556467

[CIT0015] Harvey RA, Hayden JD, Kamble PS, Bouchard JR, Huang JC. 2017. A comparison of entropy balance and probability weighting methods to generalize observational cohorts to a population: a simulation and empirical example. Pharmacoepidemiol Drug Saf. 26(4):368–377. 27859943 10.1002/pds.4121

[CIT0016] Hernandez-Aquino E, Zarco N, Casas-Grajales S, Ramos-Tovar E, Flores-Beltran RE, Arauz J, Shibayama M, Favari L, Tsutsumi V, Segovia J, et al. 2017. Naringenin prevents experimental liver fibrosis by blocking TGFbeta-Smad3 and JNK-Smad3 pathways. WJG. 23(24):4354–4368. 28706418 10.3748/wjg.v23.i24.4354 PMC5487499

[CIT0017] Hernandez-Gea V, Friedman SL. 2011. Pathogenesis of liver fibrosis. In: Abbas AK, Galli SJ, Howley PM, editors. Annual review of pathology: mechanisms of disease. New York (NY): Annual Reviews; p. 425–456. 10.1146/annurev-pathol-011110-130246 21073339

[CIT0018] Higashi T, Friedman SL, Hoshida Y. 2017. Hepatic stellate cells as key target in liver fibrosis. Adv Drug Deliv Rev. 121:27–42. 28506744 10.1016/j.addr.2017.05.007 PMC5682243

[CIT0019] Hong SH, Cha HJ, Hwang-Bo H, Kim MY, Kim SY, Ji SY, Cheong J, Park C, Lee H, Kim GY, et al. 2019. Anti-proliferative and pro-apoptotic effects of licochalcone a through ROS-mediated cell cycle arrest and apoptosis in human bladder cancer cells. Int J Mol Sci. 20(15):3820–3835. 10.3390/ijms20153820 PMC6696302 31387245

[CIT0020] Kim WS, Song HY, Han JM, Byun EB. 2019. GLM, a novel luteolin derivative, attenuates inflammatory responses in dendritic cells: therapeutic potential against ulcerative colitis. Biochem Biophys Res Commun. 518(1):87–93. 31402120 10.1016/j.bbrc.2019.08.012

[CIT0021] Kong X, Horiguchi N, Mori M, Gao B. 2012. Cytokines and STATs in liver fibrosis. Front Physiol. 3:69–75. 22493582 10.3389/fphys.2012.00069 PMC3318231

[CIT0022] Koyama Y, Brenner DA. 2015. New therapies for hepatic fibrosis. Clin and Res Hepatol Gastroenterol. 39:S75–S79. 26206573 10.1016/j.clinre.2015.06.011 PMC4734896

[CIT0023] Lakner AM, Moore CC, Gulledge AA, Schrum LW. 2010. Daily genetic profiling indicates JAK/STAT signaling promotes early hepatic stellate cell transdifferentiation. World J Gastroenterol. 16(40):5047–5056. 20976841 10.3748/wjg.v16.i40.5047 PMC2965281

[CIT0024] Li XJ, Ma QY, Jiang YM, Bai XH, Yan ZY, Liu Q, Pan QX, Liu YY, Chen JX. 2017. Xiaoyaosan exerts anxiolytic-like effects by down-regulating the TNF-α/JAK2-STAT3 pathway in the rat hippocampus. Sci Rep. 7(1):353–365. 28336920 10.1038/s41598-017-00496-y PMC5428435

[CIT0025] Liu C, Hu Y, Xu L, Liu C, Liu P. 2009. Effect of Fuzheng Huayu formula and its actions against liver fibrosis. Chin Med. 4:12–22. 19558726 10.1186/1749-8546-4-12 PMC2720970

[CIT0026] Liu K, Park C, Li S, Lee KW, Liu H, He L, Soung NK, Ahn JS, Bode AM, Dong Z, et al. 2012. Aloe-emodin suppresses prostate cancer by targeting the mTOR complex 2. Carcinogenesis. 33(7):1406–1411. 22532249 10.1093/carcin/bgs156 PMC3405653

[CIT0027] Liu Y, Wang M, Luo Y, Chen C, Lu Y, Shi Y, Tang C, Zhou Q, Zhang H, Hu Y, et al. 2017. miRNA-target network analysis identifies potential biomarkers for Traditional Chinese Medicine (TCM) syndrome development evaluation in hepatitis B caused liver cirrhosis. Sci Rep. 7(1):11054–11065. 28887510 10.1038/s41598-017-11351-5 PMC5591282

[CIT0028] Liu L, Yang J, Zu B, Wang J, Sheng K, Zhao L, Xu W. 2018. Acacetin regulated the reciprocal differentiation of Th17 cells and Treg cells and mitigated the symptoms of collagen-induced arthritis in mice. Scand J Immunol. 88(4):e12712. 30176062 10.1111/sji.12712

[CIT0029] Liver Disease Committee. 2019. Guidelines for the diagnosis and treatment of liver fibrosis with integrated traditional Chinese and Western medicine (2019 edition). Chinese J Hepatol. 27:494–504. Chinese. 10.3760/cma.j.issn.1007-3418.2019.07.005 PMC12769049 31357774

[CIT0030] Lopez-Lazaro M. 2009. Distribution and biological activities of the flavonoid luteolin. MRMC. 9(1):31–59. 10.2174/138955709787001712 19149659

[CIT0031] Massague J. 1998. TGF-beta signal transduction. Annu Rev Biochem. 67:753–791. 9759503 10.1146/annurev.biochem.67.1.753

[CIT0032] Park SJ, Sohn HY, Yoon J, Park SI. 2009. Down-regulation of FoxO-dependent c-FLIP expression mediates TRAIL-induced apoptosis in activated hepatic stellate cells. Cell Signal. 21(10):1495–1503. 19470406 10.1016/j.cellsig.2009.05.008

[CIT0033] Parvez MK, Al-Dosari MS, Alam P, Rehman M, Alajmi MF, Alqahtani AS. 2019. The anti-hepatitis B virus therapeutic potential of anthraquinones derived from *Aloe vera*. Phytother Res. 33(11):2960–2970. 31410907 10.1002/ptr.6471

[CIT0034] Sanodiya BS, Thakur GS, Baghel RK, Prasad GB, Bisen PS. 2009. *Ganoderma lucidum*: a potent pharmacological macrofungus. Curr Pharm Biotechnol. 10(8):717–742. 19939212 10.2174/138920109789978757

[CIT0035] Seki E, Schwabe RF. 2015. Hepatic inflammation and fibrosis: functional links and key pathways. Hepatology. 61(3):1066–1079. 25066777 10.1002/hep.27332 PMC4306641

[CIT0036] Shi SH, Cai YP, Cai XJ, Zheng XY, Cao DS, Ye FQ, Xiang Z. 2014. A network pharmacology approach to understanding the mechanisms of action of traditional medicine: Bushenhuoxue formula for treatment of chronic kidney disease. PLoS One. 9(3):e89123. 24598793 10.1371/journal.pone.0089123 PMC3943740

[CIT0037] Shimoi K, Okada H, Furugori M, Goda T, Takase S, Suzuki M, Hara Y, Yamamoto H, Kinae N. 1998. Intestinal absorption of luteolin and luteolin 7-O-beta-glucoside in rats and humans. FEBS Lett. 438(3):220–224. 9827549 10.1016/s0014-5793(98)01304-0

[CIT0038] Song YN, Zhang GB, Lu YY, Chen QL, Yang L, Wang ZT, Liu P, Su SB. 2016. Huangqi decoction alleviates dimethylnitrosamine-induced liver fibrosis: an analysis of bile acids metabolic mechanism. J Ethnopharmacol. 189:148–156. 27196295 10.1016/j.jep.2016.05.040

[CIT0039] Su SB, Jia W, Lu A, Li S. 2014. Evidence-based ZHENG: a traditional Chinese medicine syndrome 2013. Evid Based Complement Alternat Med. 2014:484201–484202. 24891871 10.1155/2014/484201 PMC4033416

[CIT0040] Tang LY, Heller M, Meng Z, Yu LR, Tang Y, Zhou M, Zhang YE. 2017. Transforming growth factor-β (TGF-β) directly activates the JAK1-STAT3 axis to induce hepatic fibrosis in coordination with the SMAD pathway. J Biol Chem. 292(10):4302–4312. 28154170 10.1074/jbc.M116.773085 PMC5354477

[CIT0041] Tang Y, Liao Y, Kawaguchi-Sakita N, Raut V, Fakhrejahani E, Qian N, Toi M. 2011. Sinisan, a traditional Chinese medicine, attenuates experimental chronic pancreatitis induced by trinitrobenzene sulfonic acid in rats. J Hepatobiliary Pancreat Sci. 18(4):551–558. 21234610 10.1007/s00534-010-0368-z

[CIT0042] Tian JS, Peng GJ, Wu YF, Zhou JJ, Xiang H, Gao XX, Zhou YZ, Qin XM, Du GH. 2016. A GC-MS urinary quantitative metabolomics analysis in depressed patients treated with TCM formula of Xiaoyaosan. J Chromatogr B Analyt Technol Biomed Life Sci. 1026:227–235. 10.1016/j.jchromb.2015.12.026 26733091

[CIT0043] Tsuchida T, Friedman SL. 2017. Mechanisms of hepatic stellate cell activation. Nat Rev Gastroenterol Hepatol. 14(7):397–411. 28487545 10.1038/nrgastro.2017.38

[CIT0044] Wieczorek J, Malik-Sheriff RS, Fermin Y, Grecco HE, Zamir E, Ickstadt K. 2015. Uncovering distinct protein-network topologies in heterogeneous cell populations. BMC Syst Biol. 9:24–35. 26040458 10.1186/s12918-015-0170-2 PMC4480582

[CIT0045] Wu WY, Li YD, Cui YK, Wu C, Hong YX, Li G, Wu Y, Jie LJ, Wang Y, Li GR. 2018. The natural flavone acacetin confers cardiomyocyte protection against hypoxia/reoxygenation injury via ampk-mediated activation of nrf2 signaling pathway. Front Pharmacol. 9:497–512. 29867499 10.3389/fphar.2018.00497 PMC5962741

[CIT0046] Xiao D, Zhang Y, Wang R, Fu Y, Zhou T, Diao H, Wang Z, Lin Y, Li Z, Wen L, et al. 2019. Emodin alleviates cardiac fibrosis by suppressing activation of cardiac fibroblasts via upregulating metastasis associated protein 3. Acta Pharm Sin B. 9(4):724–733. 31384533 10.1016/j.apsb.2019.04.003 PMC6664101

[CIT0047] Yan Y, Jun C, Lu Y, Jiangmei S. 2019. Combination of metformin and luteolin synergistically protects carbon tetrachloride-induced hepatotoxicity: mechanism involves antioxidant, anti-inflammatory, antiapoptotic, and Nrf2/HO-1 signaling pathway. Biofactors. 45(4):598–606. 31336028 10.1002/biof.1521

[CIT0048] Yoon YJ, Friedman SL, Lee YA. 2016. Antifibrotic therapies: where are we now? Semin Liver Dis. 36(1):87–98. 26870935 10.1055/s-0036-1571295

[CIT0049] Zhang L, Schuppan D. 2014. Traditional Chinese Medicine (TCM) for fibrotic liver disease: hope and hype. J Hepatol. 61(1):166–168. 24780816 10.1016/j.jhep.2014.03.009

[CIT0050] Zhou Y, Wu R, Cai F-F, Zhou W-J, Lu Y-Y, Zhang H, Chen Q-L, Su S-B. 2021. Xiaoyaosan decoction alleviated rat liver fibrosis via the TGFβ/Smad and Akt/FoxO3 signaling pathways based on network pharmacology analysis. J Ethnopharmacol. 264:113021–113033. 32479885 10.1016/j.jep.2020.113021

